# Identification of Biomarkers for Meat Quality in Sichuan Goats Through 4D Label-Free Quantitative Proteomics

**DOI:** 10.3390/ani15060887

**Published:** 2025-03-20

**Authors:** Rui Zhang, Mengling Xu, Rui Xu, Ting Bai, Dayu Liu, Xinhui Wang, Daodong Pan, Yin Zhang, Lin Zhang, Shifeng Pan, Jiamin Zhang

**Affiliations:** 1 Meat Processing Key Laboratory of Sichuan Province, College of Food and Biological Engineering, Chengdu University, Chengdu 610106, China; 2 College of Food Science & Engineering, Ningbo University, Ningbo 315211, China; 3 Sichuan Animal Science Academy, Chengdu 610066, China; 4 College of Veterinary Medicine, Yangzhou University, Yangzhou 225009, China

**Keywords:** goat, meat quality, candidate gene, Sichuan, local breeds, quantitative proteomics

## Abstract

The Nanjiang Yellow Goat (NJYG), Jintang Black Goat (JTBG), and Jianzhou Da’er Goat (JZDEG) are representative local goat breeds for meat production in Sichuan Province, China. This study conducted a comprehensive evaluation of the meat quality of the *longissimus dorsi* muscle of three goat breeds. Variations in meat quality were observed in terms of meat pH, color, ash and fat content, water activity, and muscle fiber structure. Quantitative proteomics analysis was employed to identify biomarkers for goat meat quality, revealing hundreds of differentially expressed proteins among three goat breeds. KEGG enrichment analysis revealed enriched pathways including oxidative phosphorylation, thermogenesis, citrate cycle (TCA cycle), fatty acid degradation and metabolism, as well as valine, leucine, and isoleucine degradation. Moreover, weighted protein co-expression network analysis and protein–protein interaction analysis uncovered valuable biomarkers, including GSTM3, NDUFS, OGDH, ACO2, HADH, ACAT1, ACADS, ACAA2, HSPG2, ITGA7, PARVB, ALDH9A1, ADH5, and LOC102190016, for assessing goat meat quality. This investigation highlighted the disparities in meat quality among local goat breeds in Sichuan, China, and provided insights into underlying biological pathways and valuable biomarkers for goat meat quality.

## Simple Summary

Sichuan Province encompasses diverse terrains, including the Qinghai–Tibet Plateau, Hengduan Mountains, Yungui Plateau, Qinba Mountains, and Sichuan Basin, impacting local goat breeds. Challenging conditions in mountainous areas like the Northern Sichuan Mountains and Western Sichuan Plateau require goats to navigate rugged terrains for foraging. Adaptations such as sturdy limbs and nimble hooves are evident in goats like the Nanjiang Yellow Goat, enabling efficient movement across complex landscapes. Contrastingly, goats in flatter regions like the Sichuan Basin exhibit a more rounded physique and shorter, robust limbs, as seen in breeds like the Jintang Black Goat and Jianzhou Da’er Goat. Exploring the diverse meat quality traits and underlying molecular mechanisms can enhance goat meat production across different environments.

## 1. Introduction

The global consumption of goat meat has significantly risen due to its distinctive nutritional advantages over conventional red meats [1]. In the past decade, China has contributed 40% to global goat meat production [2]. Recognized as a substantial protein source, goat meat is characterized by lower levels of total fat, saturated fatty acids, cholesterol, and a distinct flavor, aligning with the increasing consumer demand for health-conscious dietary choices [3]. Sichuan, located in southwestern China, is celebrated for its diverse natural geography and geomorphology, which comprises fertile plains, basins, towering mountains, and deep valleys. The fertile plains, mountainous landscapes, varied vegetation, and favorable climatic conditions in Sichuan provide a range of rearing environments for diverse goat breeds [4,5,6,7].

Proteomics technologies have been utilized to investigate molecular mechanisms underlying divergences in meat quality. The high-quality *longissimus thoracis* goat meat and low-quality external intercostals goat meat display plenty of differentially expressed proteins (DEPs), with enrichments in glycolysis and the tricarboxylic acid cycle [8]. These proteins regulate the rate and extent of pH decline in meat postmortem, thereby determining the quality of goat meat. The development of DFD (dark, firm, and dry) beef, a prototypical low-quality beef, has been linked to pathways associated with energy metabolism, cellular stress responses, and oxidoreductase activity [9], with several valuable biomarkers proposed for DFD beef [10]. Recent advancements in proteomic studies on sheep and goat meat quality have been comprehensively reviewed [11].

**Figure 1:**
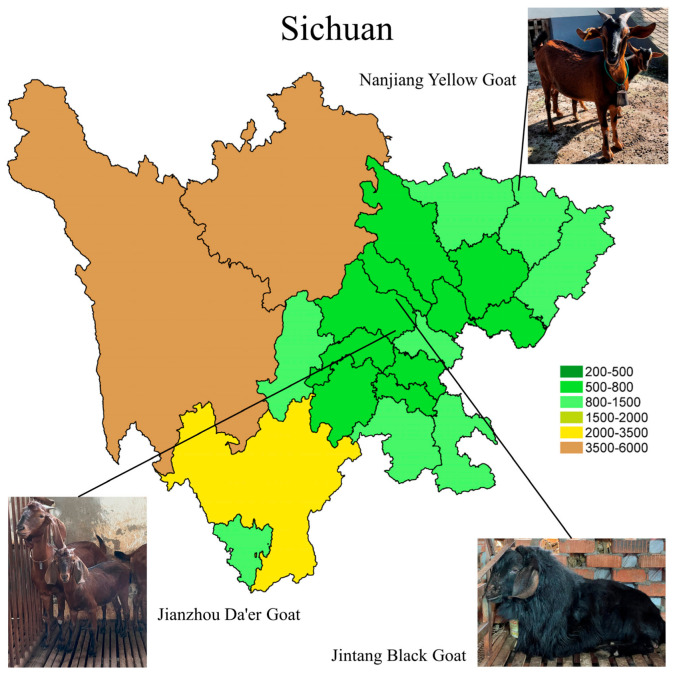
The primary habitats of the Nanjiang Yellow Goat, Jintang Black Goat, and Jianzhou Da’er Goat are depicted on a map of Sichuan, with elevations indicated by color gradients.

The Nanjiang Yellow Goat (NJYG), Jintang Black Goat (JTBG), and Jianzhou Da’er Goat (JZDEG) are renowned goat breeds for meat production in Sichuan Province. The NJYG predominantly inhabits in northern region of Sichuan, shown in Figure 1, characterized by mountainous terrain ranging from 370 to 2507 meters (m) in altitude. The JTBG is predominantly found in the western Chengdu Plain, thriving in shallow and deep hill environments with altitudes ranging from 450 to 1090 m. The JZDEG is located in the Longquan Mountain area at an altitude of around 1050 m. Several studies have examined these three goat breeds, exploring the genetic composition of the JTBG [12], growth-related gene expression of the NJYG [13], and muscle growth of the JZDEG [14]. Additionally, sausages derived from JZDEG meat show a high quality, highlighting its superior goat meat quality [15]. Nevertheless, detailed data delineating characters of meat quality from these goat breeds are currently missing. Hence, this study aims to investigate meat quality characters of the NJYG, JTBG, and JZDEG, and uncover underlying mechanisms, encompassing key pathways and potential candidate genes as biomarkers of goat meat quality.

## 2. Materials and Methods

### 2.1. Animals and Sampling

This study involved 12 male goats, with four of each breed: NJYGs with a slaughter weight of 14.2 ± 2.1 kg were sourced from Sichuan Dejian Nanjiang Huangyang Food Co., Ltd. Nanjiang, China, JTBGs with a slaughter weight of 31.9 ± 1.7 kg were obtained from Chengdu Chuanmu Black Goat Professional Cooperative, Chengdu, China and JZDEGs with a slaughter weight of 40.2 ± 5.2 kg were acquired from Sichuan Jianyang Dageda Animal Husbandry Co., Ltd, Jianyang, China. These goats were raised under a semi-grazing system, which combines free-range grazing and stall-feeding. During the grazing period, the goats had access to natural pastures. In addition to the forage from grazing, goats were supplemented with high quality feedstuffs, such as corn, wheat, soybean meal, salt, and a mineral mix. Goats aged 12 months were ethically slaughtered at local facilities after a 24 hour (h) fasting period, in accordance with the Regulation of Experimental Animals at Chengdu University (2016-4). The *longissimus dorsi* (LD) muscle was excised, with visible connective tissue and fat meticulously removed. A section was promptly frozen in liquid nitrogen and transferred to −80 °C freezer for proteomic study, while remaining samples were transported to the laboratory in ice boxes and stored at 4 °C for meat quality analysis.

### 2.2. Eating Quality

#### 2.2.1. Meat pH and Color

Meat pH was assessed at 0.5 h and 24 h after slaughter with a Testo 205 pH meter (Testo, Lenzkirch, Germany). Meat color was assessed employing a CR-10 colorimeter (Konica Minolta, Osaka, Japan) under illuminant D65, a 10° observer angle, and an 8 mm aperture. The lightness L*, redness a*, and yellowness b* of meat sample were measured at 0.5 h and 24 h after slaughter. After calibration with a standard white plate, three measurements were taken for each sample [16]. The average value and standard deviation (SD) were calculated based on four samples for each breed.

#### 2.2.2. Cooking Loss

A method adapted from Jo et al. [17] was applied to determine the cooking loss. Initially, a fresh meat sample, measuring 6 × 3 × 3 cm, was weighed as m1 and subsequently sealed in a plastic bag. The samples were cooked in water for 30 minutes (min) at 80 °C, followed by overnight cooling at 4 °C. After removing surface water, the sample weight was recorded as m2. The cooking loss value was calculated using the following equation: Cooking loss % $=\;\frac{m1-m2}{m1}\;\times \;100$. This calculated the percentage of mass loss during the cooking process of the meat sample.

#### 2.2.3. Shear Force

Shear force was measured with a TA-XT Plus texture analyzer (Stable Micro Systems, Surrey, UK) following cooking loss evaluation [18]. Meat samples were sliced into 3 cm × 1 cm × 1 cm dimensions and analyzed with a Warner-Bratzler Blade Set featuring a ‘V’ slot blade, following a pre-test rate of 2.0 mm/s, a test rate of 1.0 mm/s, and a post-test rate of 2.0 mm/s. Each sample was assessed in triplicate. The average shear force in newton (N) and SD was calculated for each breed based on four samples.

### 2.3. Proximate Analysis

Two grams of meat sample were extracted with petroleum ether solvent, evaporated to remove the solvent, and dried to determine crude lipid in accordance with the National Standard GB 5009.6-2016 [19]. Crude protein analysis was conducted based on National Standard GB 5009.5-2016 [20]. The water activity measurement involved placing five grams of meat sample in a water activity meter dish, as outlined in National Standard GB 5009.238-2016 [21]. Ash content was determined using a muffle furnace following the National Standard GB 5009.4-2010 [22]. These analyses were conducted in triplicate for each sample, with the average and SD calculated based on four samples for each breed.

### 2.4. Histology Analysis

For histological analysis, the meat sample was fixed with paraformaldehyde for paraffin embedding. The 5 μm slices perpendicular to muscle fibers were cut and stained with haematoxylin and eosin (H&E). Subsequently, images were taken using BA210 digital microscope (Motic, Xiamen, China). The diameter and area of muscle fibers were quantified utilizing ImageJ software (v. 1.53c). Each sample had ten values. The average and SD were calculated from four samples for each breed.

### 2.5. Transmission Electron Microscope

Samples were fixed using glutaraldehyde and osmium tetroxide, followed by dehydration with an acetone series and embedding in Epon 812. Semi-thin and ultra-thin sections were stained with methylene blue and uranyl acetate with lead citrate, respectively. Subsequently, images were captured using a JEM-1400 Flash transmission electron microscope (JEOL, Tokyo, Japan). For sarcomere length analysis, one hundred measurements were recorded for each sample using ImageJ software, and the average along with SD was calculated based on four samples per breed.

### 2.6. Proteomics

#### 2.6.1. Protein Extraction and Digestion

Three meat samples for each breed were used for proteomic analysis. The crushed sample was incubated in lysis solution for 5 min and subjected to ultrasonication on ice for 10 min, followed by a 20 min centrifugation at 13,000× *g* at 4 °C. The resulting supernatant was mixed with four volumes of acetone at −20 °C for 2 h. The protein pellet obtained after a second centrifugation was dried and dissolved in a buffer (8 mol/L (M) urea, 100 mM triethylammonium bicarbonate, pH 8.0). The protein solution was treated with 10 mM DTT at 56 °C for 30 min, 50 mM iodoacetamide for 30 min in darkness. The protein solution was digested with 50 times trypsin (*w*/*w*) at 37 °C for 16 h, followed by desalting using a C18 cartridge and drying with a vacuum concentrator.

#### 2.6.2. Liquid Chromatography–Tandem Mass Spectrometry (LC-MS/MS)

The digested peptides were analyzed with a nanoElute UHPLC (Bruker Daltonics, Bremen, Germany) coupled with a hybrid timsTOF Pro 2 mass spectrometer (Bruker Daltonics). Mobile phases A and B comprised 0.1% formic acid in water and 0.1% formic acid in ACN, respectively. The gradient of mobile phase B increased from 2% to 22% over 45 min, then to 35% over following 5 min, and further to 80% in 5 min, maintaining at 80% for an additional 5 min. The flow rate was set at 0.3 μL/min. The capillary voltage was set at 1400 V. The MS and MS/MS spectra were acquired in the mass range from 100 to 1700 *m*/*z*. The MS raw data underwent analysis using FragPipe (version 17.1), utilizing MSFragger for qualitative assessment and Phosopher for validation and filtering. These tools, in conjunction with the Uniprot-goat database, containing 20,425 entries, ensured a false discovery rate of <1% at both protein and peptide levels. Label-free quantitation was conducted using IonQuant. Proteins exhibiting a significant up-regulation with a fold change (FC) > 1.20 or a down-regulation with a FC < 0.83 were deemed as DEPs.

### 2.7. Bioinformatics Analysis

The functions of DEPs were analyzed with Gene Ontology (GO) database (http://geneontology.org/ accessed on 15 July 2024) and the Kyoto Encyclopedia of Genes and Genomes (KEGG) pathway database (https://www.genome.jp/kegg/ accessed on 20 August 2024). Additionally, the DAVID database (https://david.ncifcrf.gov/ accessed on 25 June 2024) was also utilized for enrichment analysis. Weighted protein co-expression network analysis (WPCNA) was performed using the Metware platform (https://cloud.metware.cn accessed on 3 July 2024). Furthermore, protein–protein interactions were visualized using the STRING-db server (http://string-db.org/ accessed on 14 July 2024).

### 2.8. Data Analysis

The data were statistically analyzed utilizing Prism GraphPad 9.0 software to conduct one-way analysis of variance (ANOVA) and Tukey’s HSD tests. The data were presented as average ± SD. A statistical significance level of *p* < 0.05 was applied in this study.

## 3. Results and Discussion

### 3.1. Quality Traits

#### 3.1.1. Eating Quality and Chemical Composition

The quality traits of NJYG, JTBG, and JZDEG LD meat are shown in Table 1. The NJYG LD meat demonstrated a lower pH_24h_ than JZDEG, while exhibiting the highest L*_24h_ and the lowest a*_24h_ and b*_24h_ among three goat breeds (*p* < 0.05). Myosin, the primary muscle protein, possesses a known isoelectric point (pI) of 5.4 [23]. Postmortem muscle pH gradually approximates meat pI, causing a decreased water retention in meat and water expulsion from meat [24]. This water increased the moisture content on meat surface and L* value. JZDEG LD muscle exhibited a higher b*_0.5h_ value than JTBG and a higher b*_24h_ value than NJYG. Consistent with previous report, meat pH significantly influences meat color [25], which is crucial in shaping consumer perceptions of meat quality as color is closely associated with meat freshness.

Furthermore, the ash content of NJYG LD meat was significantly higher than JTBG. The fat content of LD meat was significantly different from each other among three goat breeds. Fat content is a pivotal meat quality parameter and greatly influences the sensory perception of juiciness, flavor, and texture. Finally, the water activity of NJYG was significantly lower than JTBG and JZDEG and significantly influenced the juiciness and tenderness of meat. In our study, we included four goats of each of the breeds for data analysis. There was a risk that the sample might not accurately reflect the full range of variation within each breed. Furthermore, a limited number of samples could reduce the statistical power of our study. Our study might be less likely to detect true differences, even if they exist.

#### 3.1.2. Histological and Ultrastructural Analysis

**Figure 2:**
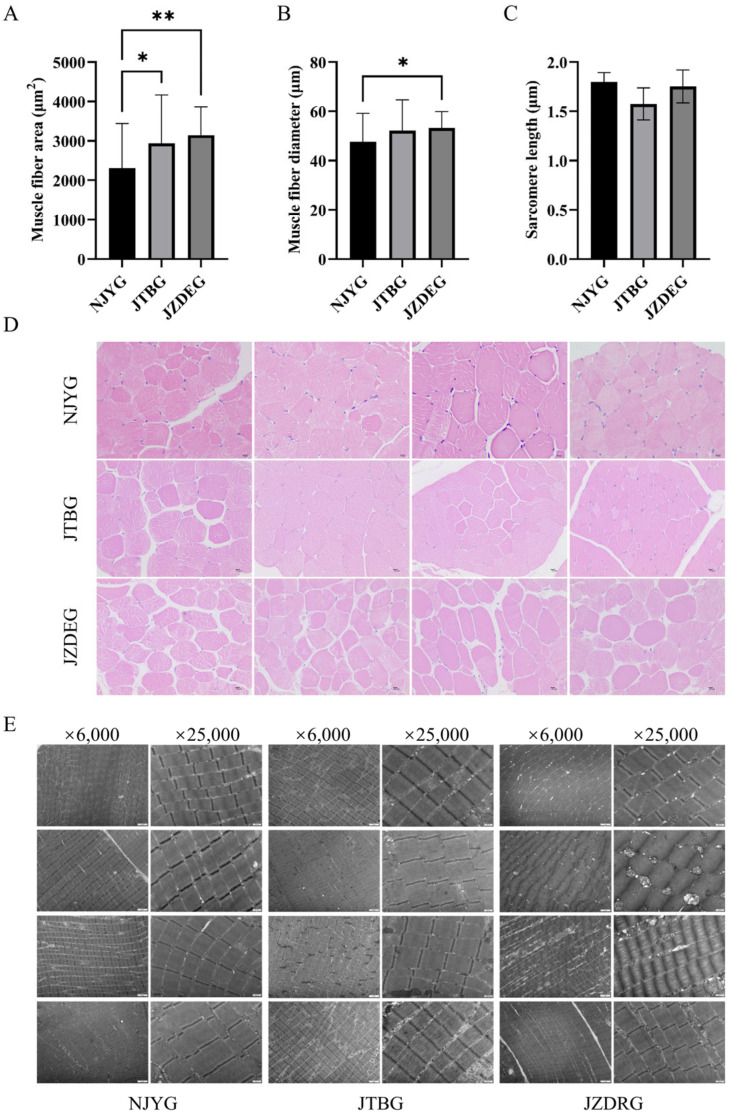
The muscle fiber area (**A**), diameter (**B**), and sarcomere length (**C**) of NJYG, JTBG, and JZDEG LD muscle. The histograms are presented as the average ± SD. The * indicates *p* < 0.05 and ** indicates *p* < 0.01. (**D**): H&E staining cross sections from LD muscle with a 10 µm scale bar. (**E**) Transmission electron micrographs of LD muscle, ×6000 bar = 2 µm and ×25,000 bar = 500 nm. Data are shown as average ± SD, n = 4.

Figure 2A,D revealed significant differences in muscle fiber area among LD muscles of three goat breeds. Specifically, NJYG LD muscle exhibited the smallest muscle fiber area, while JZDRG LD muscle displayed the largest muscle fiber area. Meanwhile, the muscle fiber diameter of NJYG LD muscle was significantly smaller than JZDEG, as seen in Figure 2B. Meat with small fiber diameter tends to be much more tender [26], although no significant differences were found for shear force among three goat breeds.

The ultrastructure of LD muscle illustrated in Figure 2E show parallel myofibrils, intact sarcomeres, and observed mitochondria. The sarcomere lengths of NJYG, JTBG, and JZDEG range from 1.37 to 2.05 µm, consistent with report about the LD muscle of the New Zealand goat [27]. Sarcomere length is a fundamental indicator of muscle tenderness; longer sarcomeres correspond to increased meat tenderness [28]. However, no significant variations in sarcomere length were observed among these three goat breeds, as seen in Figure 2C.

#### 3.1.3. Principal Component Analysis (PCA)

**Figure 3:**
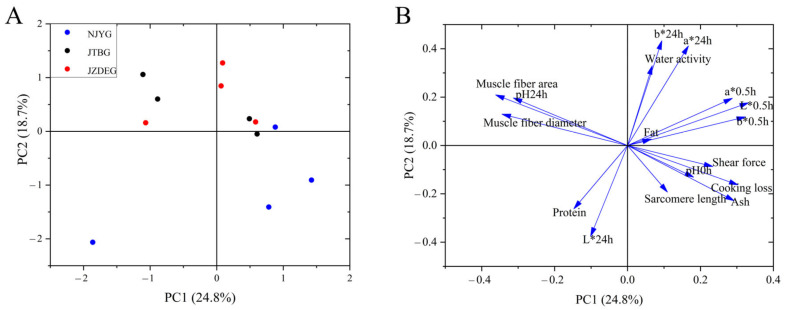
Principal component analysis of three goat meat quality using physico-chemical parameters and muscle fiber structure. PC1 vs. PC2 score–score plot (**A**) and component loading plot (**B**). Data are shown as average ± SD, n = 4.

PCA was conducted with the phenotype data mentioned above. As shown in Figure 3, NJYG exhibited a distinct separation from other two breeds and located in the low part of Figure 3A, while the divergence between JTBG and JZDEG was less discernible. The first two principal components (PC) accounted for 43.5% of total variance, with PC1 explaining 24.8% and PC2 explaining 18.7%, respectively. In the loading plot shown in Figure 3B, PC1 was characterized by muscle fiber index (fiber area and diameter), meat color (L*_0.5h_ and b*_0.5h_), and pH_24h_. Meanwhile, meat color (b*_24h_, a*_24h_, and L*_24h_), water activity, and protein content were important for PC2.

### 3.2. Proteomics Analysis

#### 3.2.1. Protein Identification and Quantification

For this evaluation, 4D label-free quantitative proteomics was employed to investigate mechanisms underlying variations in meat quality across three goat breeds. MS analysis identified a total of 496,633 spectra and 24,404 peptides. These peptides facilitated a detection of 2535 proteins, with 1984 proteins quantificated.

#### 3.2.2. DEPs Analysis

**Figure 4:**
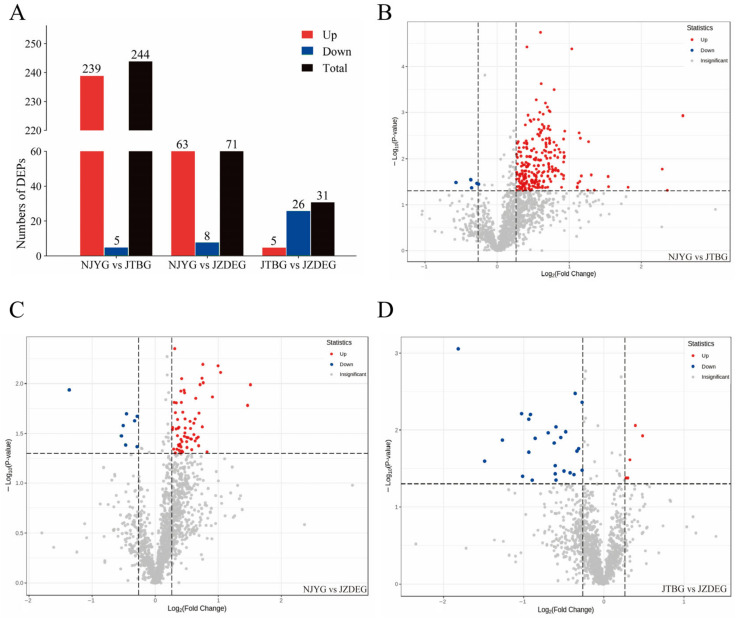
(**A**): The number of DEPs identified in this study. Volcano plot of DEPs in NJYG vs. JTBG (**B**), in NJYG vs. JZDEG (**C**), and in JTBG vs. JZDEG (**D**). The horizontal dashed line indicated position of −log_10_(0.05). The left and right vertical dashed lines indicated the positions of log_2_(0.83) and log_2_(1.2), respectively. The up-regulated or down-regulated proteins are indicated in red or blue, respectively. n = 3.

A comparative assessment between the NJYG and JTBG revealed 244 DEPs, comprising 239 up-regulated proteins and 5 down-regulated proteins in the NJYG compared to the JTBG (NJYG vs. JTBG), as shown in Figure 4A,B. This substantial number of DEPs likely stems from their distinct habitats. The NJYG thrives in the mountainous terrains of northern Sichuan, primarily subsisting on straw and shrubs, while the JTBG, situated in the Chengdu Plain, predominantly grazes on pasture grasses and legumes. In NJYG vs. JZDEG, 71 DEPs were identified, with 63 up-regulated and 8 down-regulated proteins, seen in Figure 4A,C. Furthermore, for JTBG vs. JZDEG, a total of 31 DEPs were recognized, featuring 5 up-regulated proteins and 26 down-regulated proteins, shown in Figure 4A,D.

#### 3.2.3. Enrichment Analysis

**Figure 5:**
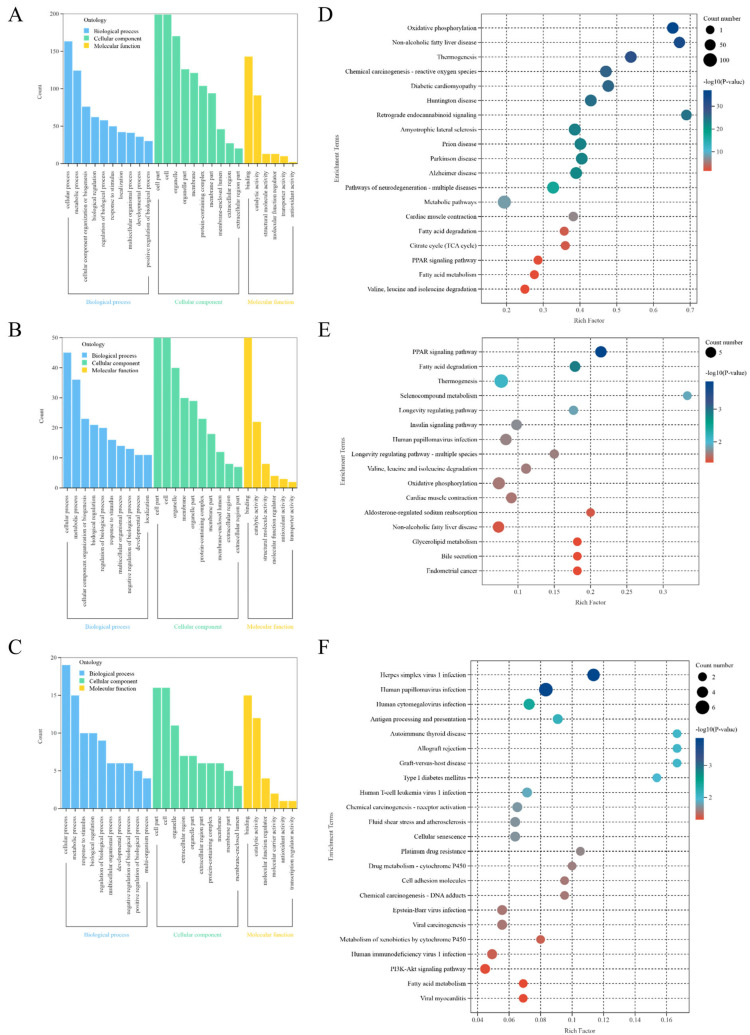
GO enrichment analysis of DEPs in NJYG vs. JTBG (**A**), NJYG vs. JZDEG (**B**), and JTBG vs. JZDEG (**C**). KEGG pathway enrichment analysis of DEPs in NJYG vs. JTBG (**D**), NJYG vs. JZDEG (**E**), and JTBG vs. JZDEG (**F**). n = 3.

GO analysis assesses proteins regarding their roles in biological processes (BP), cellular components (CC), and molecular functions (MF). The GO analysis DEPs from NJYG vs. JTBG, NJYG vs. JZDEG, and JTBG vs. JZDEG are depicted in Figure 5A–C. Notably, seven of the top ten BP ontology terms based on *p* value ranking were congruent across all three comparisons. These seven GO terms included cellular process, metabolic process, response to stimulus, biological regulation, regulation of biological process, multicellular organismal process, and developmental process. Concurrently, the GO analysis revealed common CC terms such as cell, cell part, organelle, extracellular region, organelle part, membrane, protein-containing complex, extracellular region part, membrane part, and membrane-enclosed lumen. Likewise, GO analysis highlighted the shared MF terms of binding and catalytic activity.

In NJYG vs. JTBG, 244 DEPs were notably enriched in 19 KEGG pathways, shown in Figure 5D. These pathways encompassed energy production-related processes such as oxidative phosphorylation, thermogenesis, citrate cycle (TCA cycle), and fatty acid degradation and metabolism. Additionally, enriched 19 KEGG pathways also included pathways associated with neurodegenerative diseases, such as amyotrophic lateral sclerosis, prion disease, Parkinson’s disease, and Alzheimer’s disease.

In NJYG vs. JZDEG, 71 DEPs were markedly enriched in 16 KEGG pathways, shown in Figure 5E. Notably, these pathways were linked to energy metabolism included fatty acid degradation, valine, leucine, and isoleucine degradation, thermogenesis, glycerolipid metabolism, and oxidative phosphorylation. Furthermore, enriched KEGG pathways also included the PPAR signaling pathway and insulin signaling pathway.

In JTBG vs. JZDEG, 31 DEPs were notably enriched in 23 KEGG pathways involved in disease, metabolism, and environmental information processing, shown in Figure 5F. These 23 pathways also included drug metabolism—cytochrome P450—the metabolism of xenobiotics by cytochrome P450, PI3K-Akt signaling pathway, and fatty acid metabolism.

### 3.3. WPCNA

**Figure 6:**
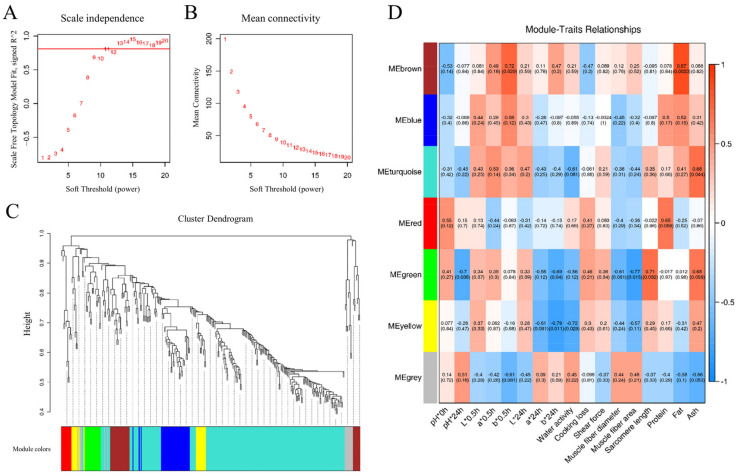
WPCNA visualization. The scale-free topology fitting index with a red line indicating the soft threshold (**A**) and mean connectivity (**B**). (**C**) Clustering dendrogram of proteins and module division. (**D**) The correlative diagram of modules and goat meat phenotypes. Pearson’s coefficients for correlations are indicated in the square grid, with *p* values in parentheses. n = 3.

WPCNA characterize co-expressed DEPs into several phenotype-related modules [29]. The soft threshold power of β = 11 was determined based on the scale-free fit index and mean connectivity to establish the WPCNA network, shown in Figure 6A,B. Subsequently, hierarchical clustering of strongly co-expressed proteins facilitated the creation of a cluster dendrogram, identifying seven distinct module elements (MEs) represented by different colors, as illustrated in Figure 6C.

The correlations between seven MEs and goat meat quality parameters were visually represented in a heatmap, shown in Figure 6D. Specifically, the brown ME (MEbrown) exhibited positive correlations with b*_0.5h_ (r = 0.72, *p* = 0.029) and fat content (r = 0.87, *p* = 0.0023). MEturquoise showed a positive correlation with ash content (r = 0.68, *p* = 0.044). Furthermore, MEgreen demonstrated negative correlations with pH*_24h_ (r = −0.7, *p* = 0.036), b*_24h_ (r = −0.69, *p* = 0.04), and muscle fiber area (r = −0.77, *p* = 0.015), while exhibiting a positive correlation with sarcomere length (r = 0.71, *p* = 0.032). MEyellow displayed negative correlations with b*_24h_ (r = −0.79, *p* = 0.011) and water activity (r = −0.72, *p* = 0.029). To delve deeper into protein networks within each ME associated with goat meat quality, a protein–protein interaction network (PPI) analysis focused on these four modules—MEbrown, MEturquoise, MEgreen, and MEyellow.

### 3.4. PPI Analysis

**Figure 7:**
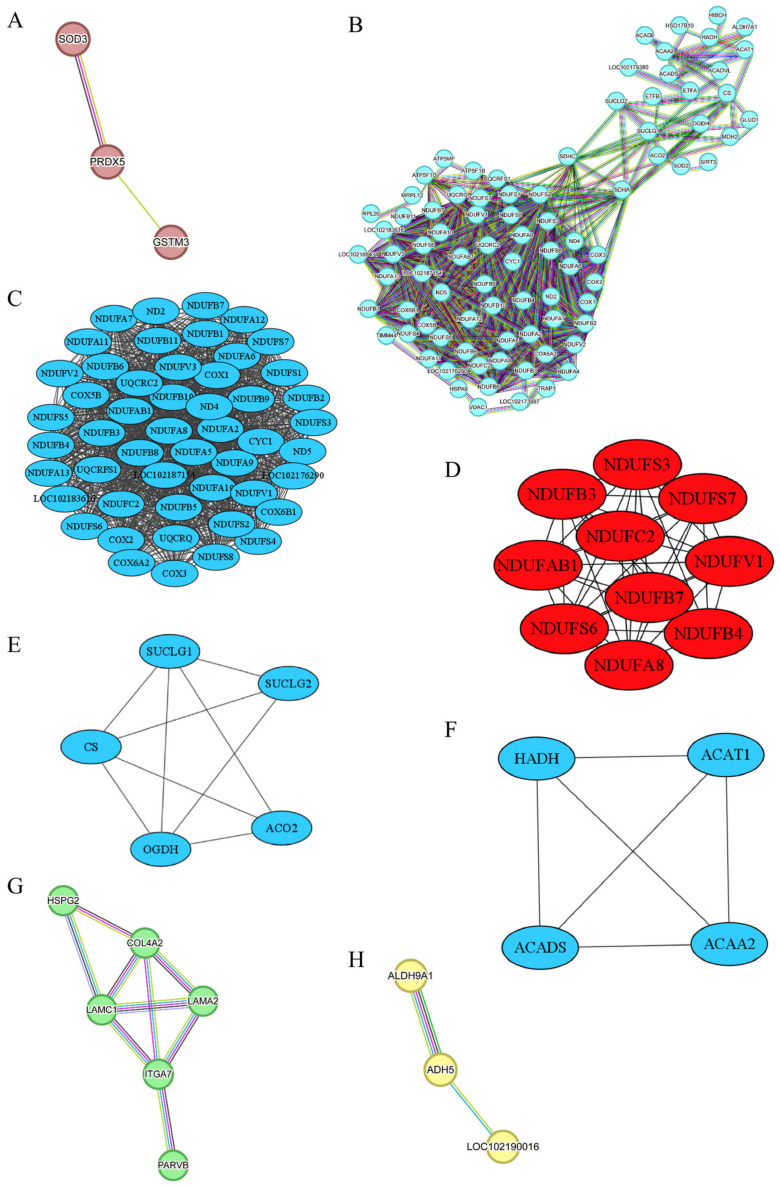
PPI networks for MEbrown (**A**), MEturquoise (**B**), sub-network B-I (**C**), and its ten hub proteins (**D**), sub-network B-II (**E**), sub-network B-III (**F**), MEgreen (**G**), and MEyellow (**H**). The color coding of the lines in the STRING was categorized into three sections: known interactions, predicted interactions, and others. In the known interactions, the sky blue line represented curated database interactions, while the purple line signified experimentally determined interactions. For the predicted interactions, green indicated gene neighborhood associations, red indicated gene fusions, and dark blue indicated gene co-occurrence. Other interactions are depicted as medium yellow for text mining, black for co-expression, and light blue for protein homology.

Figure 7A demonstrates the PPI analysis based on MEbrown, indicating a significant enrichment with *p* < 0.033. Within MEbrown, three proteins—Glutathione S-Transferase Mu 3 (GSTM3), Superoxide Dismutase 3 (SOD3), and Peroxiredoxin 5 (PRDX5)—exhibited direct interactions. GSTM3 plays a crucial role in upholding cellular homeostasis, shielding against external toxins, and safeguarding cells from oxidative stress. It serves as a potential biomarker for sheep meat tenderness, displaying a higher expression in tender sheep meat [30]. Moreover, GSTM1, a member of the GSTM family, demonstrates a positive association with beef tenderness [31]. Although our research found no significant difference in the tenderness of goat meat, we observed a higher abundance of GSTM3 in goat meat with a higher fat content. Although the significance of fat content in determining meat tenderness remains debated [32], multiple works have pointed out the role of fat content in destemming the meat tenderness [32,33,34,35]. As mentioned above, MEbrown was also linked with b*_0.5h_. One previous study has shed lights on GSTM1, GSTM3, and GSTM5 as candidate biomarkers for sheep meat color due to their involvement in oxidative stress and cell redox homeostasis [36]. Our study further underscored the impact of GSTM3 on meat color, although GSTM3 is not traditionally regarded as a biomarker for meat color. In addition to GSTM3, our research identifies anti-oxidative stress genes SOD3 and PRDX5 as potential candidate genes for goat meat color and fat content. SOD3, found exclusively in extracellular spaces, shields cells and tissues from oxidative stress by removing superoxide radicals [37]. In contrast, PRDX5 is present in various subcellular compartments, including mitochondria, peroxisomes, cytosol, and nucleus, combating peroxide attacks as a cytoprotective antioxidant enzyme [38]. Several Peroxiredoxin family members, such as PRDX1, PRDX2, PRDX3, and PRDX6, have been linked to beef color [39,40]. As shown in Table 2, our investigation revealed a lower abundance of GSTM3, SOD3, and PRDX5 in JTBG LD meat characterized by higher a*_24h_ and b*_24h_, confirming the influence of cellular redox status and related genes on meat color.

The PPI network analysis of DEPs derived from the MEturquoise module revealed a significant enrichment with *p* < 1.0 × 10^−16^, as depicted in Figure 7B. To simplify its complexity, we identified three distinct sub-networks via the MCODE clustering method [41]. Furthermore, cytohubba analyzed the sub-network B-I [42], seen in Figure 7C, and extracted ten hub proteins, namely NADH:Ubiquinone Oxidoreductase subunit A8 (NDUFA8), NADH:Ubiquinone Oxidoreductase subunit AB1 (NDUFAB1), NADH:Ubiquinone Oxidoreductase subunit B3 (NDUFB3), NDUFB4, NDUFB7, NADH:Ubiquinone Oxidoreductase subunit C2 (NDUFC2), NADH:Ubiquinone Oxidoreductase Core subunit S3 (NDUFS3), NDUFS6, NDUFS7, and NADH:Ubiquinone Oxidoreductase Core subunit V1 (NDUFV1), exhibiting high connectivity, shown in Figure 7D. These ten genes encode subunits of NADH–ubiquinone oxidoreductase (Complex I), a pivotal component in the mitochondrial electron transport chain responsible for electron transfer from NADH to ubiquinone and promoting ATP production through oxidative phosphorylation [43]. As indicated in Table 2, Complex I-associated DEPs exhibited higher levels in NJYG compared to JTBG. Mitochondrial reactive oxygen species (ROS) primarily stem from the activity of Complex I and Complex III [44]. Disruption or inhibition of the interaction between Complex I and Complex III enhances ROS generation [45], which leads to fragmentation and structural changes in protein structure and impacts meat quality attributes such as water-holding capacity, tenderness, and gelation function [46]. The increased L* caused by a low water-holding capacity might reflect, to some extent, the impacts of ROS on goat meat quality [47]. Our work indicated that these ten hub genes could be potential biomarkers for meat color, which show divergences among the three goat breeds investigated.

As shown in Table 1, the ash content was significantly higher in NJYG than JTBG and indicates a higher mineral content in NJYG LD meat. Mitochondrial metabolome studies have revealed the critical role of mineral homeostasis in mitochondrial activity [48]. Specifically, mitochondrial accumulation of calcium ions promotes the activity of TCA cycle enzymes, thereby increasing oxidative phosphorylation and ATP synthesis [49]. Thus, a higher ash content could support a more active mitochondrial function.

The sub-network B-II included five DEPs, shown in Figure 7E. Succinate-CoA Ligase GDP/ADP-Forming Subunit alpha (SUCLG1) and Succinate-CoA Ligase GDP-Forming Subunit beta (SUCLG2) encode subunits of succinyl-CoA synthetase crucial for succinyl-CoA and succinate formation and ATP production in cellular energy metabolism. Oxoglutarate Dehydrogenase (OGDH) encodes a subunit of the 2-oxoglutarate dehydrogenase complex involved in TCA cycle. Citrate Synthase (CS) synthesizes citrate from oxaloacetate and acetyl-coenzyme A. Aconitase 2 (ACO2) enables the interconversion from citrate to isocitrate within TCA cycle and exhibited the highest abundance in NJYG, shown in Table 2. Mitochondrial metabolism induces a pH decline and promotes the transformation from muscle to meat and meat quality development postmortem [50]. These five genes in TCA cycle had a higher abundance in NJYG than JTBG shown in Table 2 and might contribute to the fast pH decline, from 6.65 to 5.60, in NJYG within 24 h postmortem, which could lead to a low protease activity and high shear force [51]. Furthermore, OGDH has shown an association with beef tenderness and potentially acts as a meat tenderness biomarker [52].

As shown in Figure 7F, the sub-network B-III comprised four DEPs, all associated with fatty acid metabolic enzymes, namely Hydroxyacyl-CoA Dehydrogenase (HADH), Acyl-CoA Dehydrogenase Short Chain (ACADS), Acetyl-CoA Acetyltransferase 1 (ACAT1), and Acetyl-CoA Acyltransferase 2 (ACAA2). These proteins play critical roles in fatty acid breakdown through beta-oxidation, a key process in energy production and lipid metabolism. These four genes have been reported to positively contribute to fat deposit in goat and ACADS is a candidate gene for intramuscular fat deposits [53]. In line with this report, NJYG had a higher abundance of these four proteins and a higher level of fat content compared to JTBG. Thus, HADH, ACAT1, ACADS, and ACAA2 might serve as candidate biomarkers for fat content in goat meat.

The PPI network analysis based on the MEgreen module revealed a significant enrichment with *p* < 2.39 × 10^−12^, highlighting 6 DEPs that exhibited strong interactions: Heparan Sulfate Proteoglycan 2 (HSPG2), Integrin α7 (ITGA7), Parvin beta (PARVB), Laminin Chain α1 (LAMC1), Laminin Subunit α2 (LAMA2), and Collagen type IV α2 chain (COL4A2), as illustrated in Figure 7G. HSPG2 encodes Perlecan, a major heparan sulfate proteoglycan crucial for basement membrane and extracellular matrix integrity. ITGA7, a cell surface receptor in skeletal muscle, mediates connections between the extracellular matrix and the internal actin cytoskeleton, mainly concentrated at muscle–tendon junctions in postnatal muscles [54]. Extensive expression of ITGA7 results in stable adhesion of myofiber to extracellular matrix [55]. PARVB plays a key role in cytoskeleton organization and cell adhesion. LAMC1, LAMA2, and COL4A2 are vital basement membrane proteins that indirectly impact muscle structure and integrity. Basement membranes and the extracellular matrix constitute the intramuscular connective tissues critical for meat tenderness. As muscles grow, the heightened integrity and complexity of intramuscular connective tissues enhance meat mechanical strength, leading to tougher meat [56]. MEgreen module was significantly associated with muscle fiber area, sarcomere length, and meat color. Our findings suggest that up-regulation of cell adhesion related proteins in NJYG could promote extensive connections among muscle fibers and their adhesion to extracellular matrix, potentially enhancing the uniformity and texture of meat in NJYG. In summary, the interplay and functionality of these genes related to basement membranes, extracellular matrix components, muscle structure, and cytoskeleton organization play pivotal roles in defining meat quality attributes including tenderness, texture, and muscle mechanical strength.

The PPI network analysis of the MEyellow module revealed an enrichment with *p* < 0.108, showcasing three genes: Aldehyde Dehydrogenase 9 Family Member A1 (ALDH9A1), Alcohol Dehydrogenase 5 (ADH5), and LOC102190016, as depicted in Figure 7H. ALDH9A1 detoxifies reactive aldehydes generated during the synthesis of various cellular compounds. It has been documented that ALDH9A1 overexpression inhibits intracellular triglyceride synthesis in cattle LD muscle [57]. ADH5 plays a crucial role in regulating intramuscular fatty acid content and composition in pork [58]. LOC102190016, also known as Glutathione S-Transferase Omega-1, possesses glutathione-dependent thiol transferase activity and is pivotal for maintaining cellular redox homeostasis [59]. Glutathione S-Transferase and Glutathione S-Transferase Omega-1-like genes are reported to link with mutton tenderness [30]. In the current study, these three genes had a higher abundance in the NJYG compared to JZDEG, as shown in Table 2. The divergence of meat quality between the NJYG and JZDEG mainly lay in meat color, pH, and fat content. Furthermore, MEyellow was associated with meat color. Thus, our study suggested ALDH9A1, ADH5, and LOC102190016 as potential biomarkers for goat meat color.

## 4. Conclusions

Goat meat is highly favored in Sichuan, particularly during winter. This study conducted a comprehensive assessment of meat quality of three representative meat goat breeds, NJYG, JTBG, and JZDEG, in Sichuan, China. The variations in meat quality of three goat breeds encompassed parameters such as pH, meat color, water activity, ash, fat content, and muscle fiber structure. The quality of goat meat is influenced by both their habitat and genetic background. Utilizing a proteomic approach, this study identified that the divergence in quality traits was mainly attributed to proteins related to energy production and metabolism, fatty acid degradation and metabolism, as well as valine, leucine, and isoleucine degradation. Multiple biomarkers were identified for key aspects of goat meat quality, including meat color, fat content, tenderness, and muscle fiber structure. These biomarkers can be utilized to predict adult meat quality by integrating biomarker profiling with early-life tissue sampling, thereby reducing reliance on post-mortem evaluations and shortening generational intervals. Moreover, the combination of biomarker profiling with genetic markers, such as SNPs or haplotypes, can enhance the accuracy of genomic selection programs aimed at breeding goats with desired meat quality attributes. This study provides valuable insights into underlying mechanisms for diversity in meat quality among Sichuan goat breeds, highlighting potential biomarkers that could be applied in the meat industry and genetic selection processes. Nevertheless, given the limited sample size in the present study, further research with a larger sample size is necessary to validate these conclusions and discover additional valuable biomarkers related to the sensory and nutritional attributes of goat meat.

## Figures and Tables

**Table 1:** Quality traits of *longissimus dorsi* meat.

Parameters	NJYG	JTBG	JZDEG
pH_0.5h_	6.65 ± 0.19	6.59 ± 0.26	6.55 ± 0.38
L*_0.5h_	34.68 ± 4.47	34.64 ± 0.89	33.65 ± 2.02
a*_0.5h_	16.10 ± 1.82	15.40 ± 0.62	16.15 ± 1.09
b*_0.5h_	4.22 ± 0.72 ^ab^	3.80 ± 0.17 ^b^	4.70 ± 0.70 ^a^
pH_24h_	5.60 ± 0.06 ^b^	5.74 ± 0.20 ^ab^	5.85 ± 0.32 ^a^
L*_24h_	44.61 ± 5.15 ^a^	39.36 ± 2.54 ^b^	39.60 ± 2.91 ^b^
a*_24h_	12.43 ± 5.31 ^b^	17.73 ± 2.39 ^a^	17.35 ± 1.09 ^a^
b*_24h_	4.44 ± 4.39 ^b^	7.70 ± 2.79 ^a^	10.50 ± 2.00 ^a^
Cooking loss (%)	28.80 ± 3.90	30.00 ± 7.23	22.11 ± 8.26
Shear force (N)	122.70 ± 10.36	93.27 ± 13.49	100.13 ± 44.70
Chemical composition			
Ash (%)	1.50 ± 0.12 ^a^	1.36 ± 0.11 ^b^	1.43 ± 0.05 ^ab^
Fat (%)	3.95 ± 0.17 ^b^	3.64 ± 0.16 ^c^	4.25 ± 0.06 ^a^
Protein (%)	24.48 ± 4.55	21.72 ± 2.56	24.61 ± 3.89
Water activity (%)	0.95 ± 0.00 ^b^	0.98 ± 0.01 ^a^	0.97 ± 0.20 ^a^

a–c Averages sharing the same letter were not significantly different (*p* < 0.05).

**Table 2:** Relative abundance of proteins in PPI.

Module		Protein	NJYG	JTBG	JZDEG	FC		
						NJYG vs. JTBG	NJYG vs. JZDEG	JTBG vs. JZDEG
MEbrown		GSTM3	6.386	5.293	10.106			0.524 *
		SOD3	0.545	0.397	0.612			0.649 *
		PRDX5	1.548	1.195	1.824	1.295 *		
MEturquoise	B-I	NDUFS3	5.241	3.150	3.944	1.664 **		
		NDUFA8	3.870	2.421	2.895	1.598 **		
		NDUFS6	3.030	1.903	2.362	1.592 ***		
		NDUFS7	8.375	5.761	6.724	1.454 *		
		NDUFAB1	4.332	2.275	3.282	1.904 **		
		NDUFV1	3.420	2.215	2.745	1.544 **		
		NDUFB7	2.797	1.705	2.134	1.641 **		
		NDUFC2	5.413	3.325	4.033	1.628 **		
		NDUFB4	4.523	2.750	3.262	1.644 **	1.386 *	
		NDUFB3	4.786	3.090	3.697	1.549 **		
	B-II	SUCLG2	3.575	1.654	2.061	2.161 *		
		SUCLG1	8.394	6.084	6.824	1.380 *		
		OGDH	5.607	4.253	4.882	1.319 *		
		ACO2	15.219	8.030	10.182	1.895 *	1.495 *	
		CS	16.078	10.818	12.348	1.486 *		
	B-III	HADH	4.330	2.654	3.386	1.632 *		
		ACAT1	12.509	5.618	7.839	2.227 **		
		ACADS	2.219	0.763	1.647	2.908 *		
		ACAA2	2.785	1.239	1.609	2.248 *		
MEgreen		HSPG2	7.333	5.765	5.913	1.272 *	1.240 **	
		COL4A2	13.659	8.980	10.846	1.521 *		
		LAMC1	3.253	2.790	2.638		1.233 *	
		LAMA2	10.567	8.793	8.141		1.298 *	
		ITGA7	0.532	0.414	0.457	1.285 *		
		PARVB	0.558	0.370	0.406	1.508 **	1.374 *	
MEyellow		ALDH9A1	1.052	0.747	0.650	1.409 *	1.619 *	
		ADH5	2.931	2.566	2.419		1.211 *	
		LOC102190016	2.076	1.921	1.294		1.605 *	

The *, **, and *** represented significance levels *p* < 0.05, *p* < 0.01, and *p* < 0.001, respectively.

## Data Availability

The proteomics data have been archived in the China National GeneBank (https://db.cngb.org, accessed on 8 September 2024) with the accession number CNP0006223. Other data will be available on request to corresponding authors.
